# Unusual Localization of Adenoid Cystic Carcinoma: A Case Report of Nasopharyngeal Involvement

**DOI:** 10.7759/cureus.95678

**Published:** 2025-10-29

**Authors:** Nourelhouda Mouhib, Soumiya Samba, Anass Haloui, Nassira Karich, Amal Bennani, Ahmed BenSghier, Soufiane Berhili, Mohamed Moukhlissi, Loubna Mezouar

**Affiliations:** 1 Department of Radiation Oncology, Faculty of Medicine and Pharmacy, Centre Hospitalier Universitaire Mohammed VI, Oujda, MAR; 2 Department of Pathology, Mohammed VI University Hospital/Faculty of Medicine, Mohammed 1st University, Oujda, MAR

**Keywords:** adenoid cystic carcinoma (acc), lung metastatic adenoid cystic carcinoma, nasopharyngeal carcinoma, radiation therapy, unusual presentation

## Abstract

Adenoid cystic carcinoma is a malignant tumor of the salivary glands. Its presence in the nasopharynx is rare. It grows slowly but spreads locally and invades nerves.

We present the case of a patient aged 62, a smoker, with adenoid cystic carcinoma of the nasopharynx, found first by otorhinolaryngological-specific symptoms and then by neurologic manifestations. MRI showed a tumor in the left posterosuperior wall of the nasopharynx with intracranial extension. Biopsy confirmed the diagnosis; therefore, the patient received combined chemoradiotherapy. The progression of the disease is marked by a distant recurrence after one year of follow-up. The challenge with this rare tumor entity is both diagnostic and therapeutic.

## Introduction

Adenoid cystic carcinoma is a slow-growing yet aggressive malignant tumor with a high propensity for local invasion, perineural spread, and potential intracranial extension. While it most commonly arises in the salivary glands, its occurrence in the nasopharynx is exceptionally rare, accounting for 0.13-0.48% of all malignant nasopharyngeal tumors, and it may involve cervical lymph nodes [1]. Moreover, it differs from the classic undifferentiated squamous cell carcinoma by its neurotropic behavior, lack of association with the Epstein-Barr virus, and characteristic glandular architecture. The diagnosis is based on histological and immunohistochemical examination. The primary treatment is mainly surgical, often followed by radiotherapy. However, when there is an intracranial involvement, surgery may become highly complex or even impossible. The prognosis of adenoid cystic carcinoma remains variable, depending on multiple factors such as tumor stage, cranial nerve involvement, the completeness of surgical margins, and the presence of distant metastases. We report a rare case of adenoid cystic carcinoma of the nasopharynx, aiming to underline both the exceptional nature of this tumor location and the diagnostic and therapeutic challenges associated with its advanced, unresectable stage.

## Case presentation

The patient, a 62-year-old chronic smoker (35 pack-years) and former alcoholic, presented with a one-year history of headaches, dizziness, and left-sided hearing loss (hypoacusis). Clinical examination revealed a bulging of the Rosenmüller fossa on the left, a 4 cm left lateral cervical lymphadenopathy, and cranial nerve involvement characterized by hypoesthesia in the V1, V2, and V3 territories and left abducens nerve palsy.

**Figure 1:**
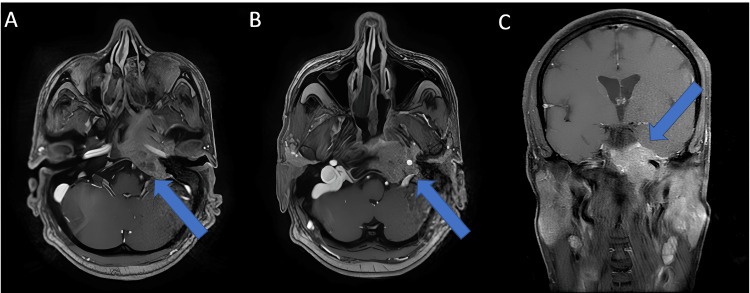
Angio-MRI of the nasopharynx in contrast-enhanced T1 sequence A: axial view showing a soft-tissue lesion (arrow) centered on the left posterosuperior wall of the nasopharynx, measuring approximately 53 × 42 mm, invading the parapharyngeal space and the left infratemporal fossa; B: axial view showing the lesion (arrow) encasing the left internal carotid artery; C: coronal view demonstrating the intracranial extension of the tumor (arrow).

MRI angiography identified a tissue mass located at the upper back wall of the left side of the nasopharynx. The lesion appeared isointense on T1-weighted images and showed contrast enhancement after gadolinium injection, measuring 53 × 42 mm. The tumor infiltrated the posterior cranial fossa and the temporal region, with lysis of the left petrous apex, clivus, and sphenoid sinus. It also invaded the jugular bulb, encasing the left internal carotid artery (Figure 1).

**Figure 2:**
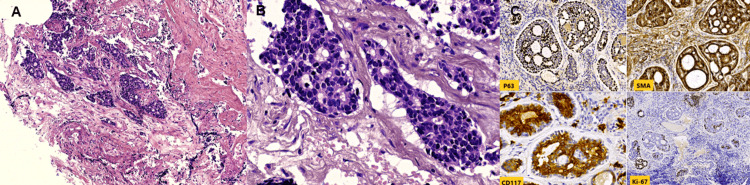
Immunohistochemical analysis findings A: low-power view showing the infiltration of nasopharyngeal mucosa by a tumoral proliferation displaying a cribriform pattern (hematoxylin-eosin-saffron coloration, magnification X10); B: high-power view showing the characteristic “Swiss cheese” appearance, with pseudocysts and peripheral cleft-like spaces filled with myxoid material, and true glandular lumina lined by a biphasic epithelium made of ductal and myoepithelial cells. (hematoxylin-eosin-saffron coloration, magnification x40); C: the myoepithelial layer was stained by P63 and SMA, whereas the ductal cells were positive for CD117. The proliferation index estimated by Ki-67 was 25%.

A nasopharyngeal biopsy was scheduled for the day after the MRI and was subsequently performed. Histopathological examination revealed a carcinomatous proliferation with cribriform and tubular architecture, including cystically dilated tubular structures. Immunohistochemical analysis showed positive staining for CD117, AML, and p63, consistent with adenoid cystic carcinoma (Figure 2).

Following a staging workup, including a thoracoabdominopelvic CT scan, the tumor was classified as T4N1M0 according to the American Joint Committee on Cancer (AJCC) 8th edition for nasopharyngeal tumors, indicating an advanced local tumor with regional lymph node involvement but no evidence of distant metastasis.

After discussion in a multidisciplinary tumor board meeting, the patient received external volumetric modulated arc therapy (VMAT) radiotherapy, with a total dose of 70 Gy delivered in 35 fractions, related to sensitizing chemotherapy consisting of six weekly cycles of CDDP at 35 mg/m² over 49 days.

**Figure 3:**
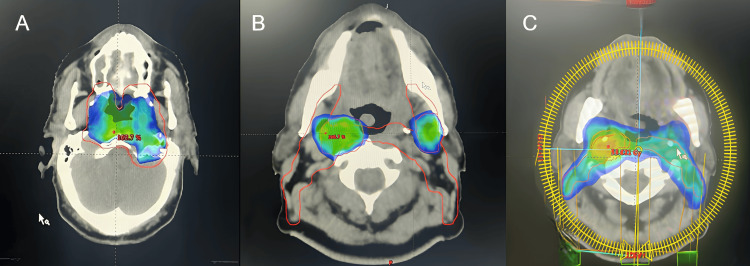
The patient underwent an external volumetric modulated arc therapy at a total dose of 70 Gy in 35 fractions of 2 Gy, five days per week A: an axial image showing the 50 Gy dose distribution at the level of the nasopharynx; B, C: an axial image showing the 70 Gy and 50 Gy dose distribution at lymphadenopathy.

Radiotherapy was administered sequentially. The first phase consisted of a dose of 50 Gy delivered to the primary gross tumor volume (GTV), clinical target volume (CTV), and prophylactic cervical lymph node regions. This was followed by a second phase of 20 Gy directed at the tumor GTV and involved lymph nodes, following reassessment and adaptation of the target volume. The treatment approach was carefully planned to respect dose constraints for organs at risk, particularly the brainstem, for which the accepted dose constraint was 56 Gy, while the spinal cord was limited to < 45 Gy, and the optic nerves and chiasm to < 54 Gy (Figure 3). Acute toxicities included grade 2 mucositis and dermatitis, along with mild weight loss.

**Figure 4:**
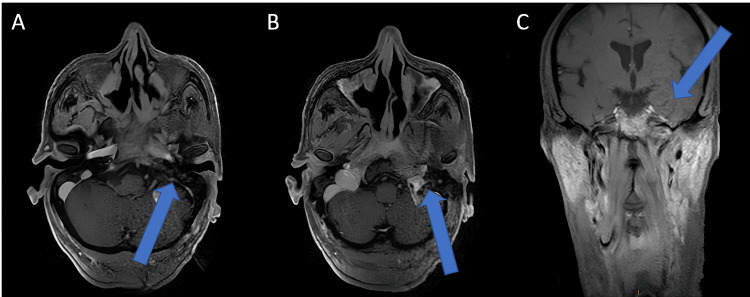
Post-treatment contrast-enhanced T1 sequence angio-MRI of the nasopharynx A, B: axial views showing a reduction in tumor size (arrow) to approximately 30 × 26 mm, with disappearance of invasion of the left infratemporal fossa; C: coronal view showing resolution of the intracranial extension (arrow).

At six months of follow-up, there was a marked improvement in symptoms, complete regression of the cervical lymphadenopathy, and approximately a 60% radiological response on follow-up cervico-facial MRI. The lesion measured 30 × 26 mm, with resolution of intracranial extension and disappearance of invasion into the sphenoidal sinus, left cavernous sinus, and left infratemporal fossa (Figure 4).

**Figure 5:**
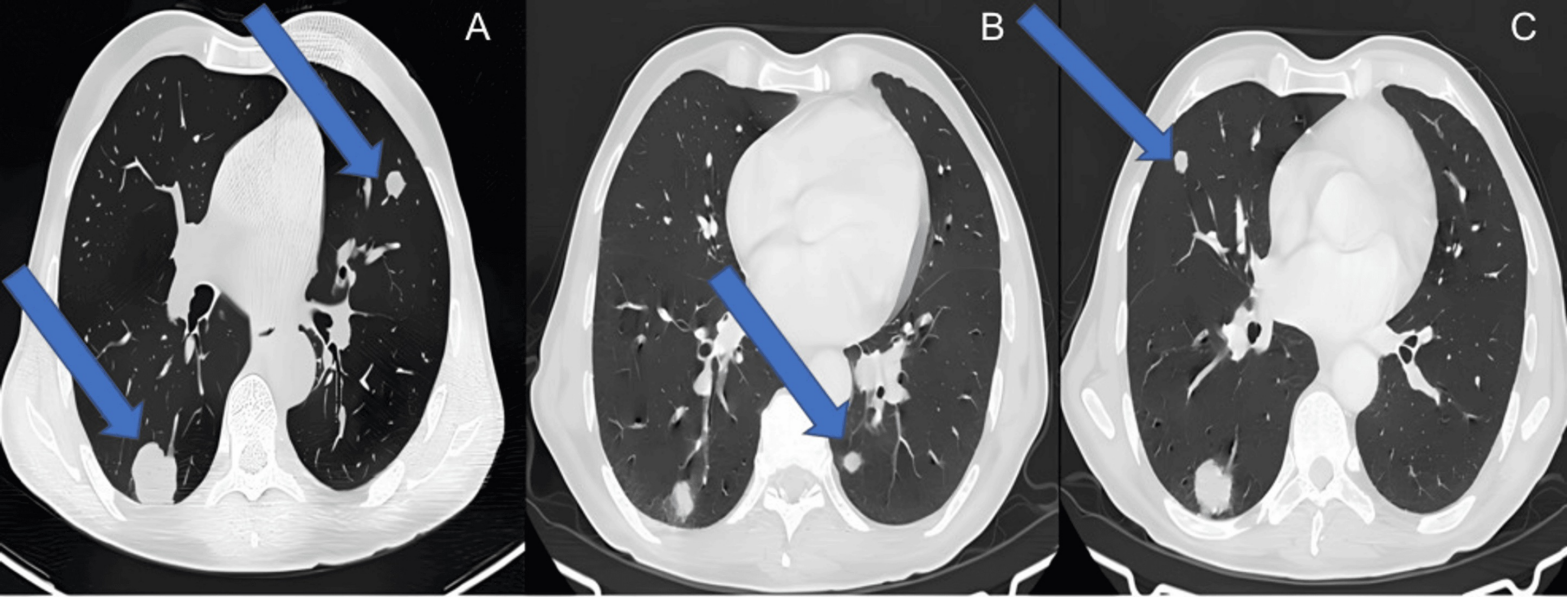
Axial slices of a contrast-enhanced chest CT scan in lung window A, B, C: axial slices of a contrast-enhanced chest CT scan in lung window, demonstrating multiple bilateral pulmonary nodules suggestive of secondary lesions (arrows). The largest nodule, located in the right lower lobe and measuring 30 × 28 mm, is shown in A.

However, at 12 months, multiple bilateral lung metastases were detected, with the largest nodule measuring 30 × 28 mm in the right lower lobe. Palliative chemotherapy was subsequently initiated (Figure 5).

## Discussion

Adenoid cystic carcinoma, also known as cylindroma, is a malignant tumor that usually affects the salivary glands. Its occurrence in the nasopharynx is rare, accounting for only 0.13 to 4% of nasopharyngeal carcinomas [2].

It is a slow-growing cancer, which often leads to delayed diagnosis and treatment. The interval between the onset of the disease and the appearance of the first symptoms is estimated to range from two to five years [3,4]. This carcinoma is slightly predominant in females and most commonly affects patients between 30 and 60 years [2].

The most frequently reported symptoms include epistaxis (nosebleeds), progressive nasal obstruction, hearing loss, and tinnitus. When the tumor extends to the skull base, additional symptoms may develop, such as oculomotor disturbances, diplopia (double vision), facial neuralgia, and dysfunction of cranial nerves IX, X, XI, and XII, and, rarely, Horner’s syndrome [5].

Nasopharyngeal adenoid cystic carcinoma is characterized by high local aggressiveness. It often spreads along nerves (perineural invasion) and can involve nearby cranial nerves, sometimes extending into the orbit or the skull base [2,6,7]. At the time of diagnosis, cervical lymph node involvement is present in 30% of cases, although less frequent distant pulmonary metastases can occur [8]. Interestingly, the rate of distant metastases appears to be similar to that seen in other nasopharyngeal cancers [7].

Histologically, adenoid cystic carcinoma tumors are classified into three main patterns. The tubular pattern is the most common, seen in approximately 50% of cases. The cribriform form, observed in about 30% of cases, tends to show more extensive local infiltration and is associated with a high recurrence rate, reported to be as high as 90% [2]. The solid variant is the rarest, accounting for roughly 10% of cases, and is considered the most aggressive due to its strong tendency to metastasize [9].

In our patient, histological examination of the tumor biopsy revealed a combination of cribriform and tubular growth patterns, along with marked locoregional infiltration, but no evidence of distant metastasis at the time of diagnosis. Immunohistochemical analysis played a key role in confirming the diagnosis of nasopharyngeal adenoid cystic carcinoma [10]. In this case, the diagnosis showed positive staining for p63, AML, and CD117. Additionally, the Ki-67 proliferation index, estimated at 25%, indicates moderate proliferative activity, suggesting a potentially significant biological aggressiveness. Nasopharyngeal MRI is essential for accurate tumor mapping, particularly for assessing perineural spread, while CT scanning provides better evaluation of bone involvement [1,2].

Surgical resection remains the standard treatment for nasopharyngeal adenoid cystic carcinoma. However, complete removal is often challenging or even impossible due to the complex anatomy of the nasopharynx and the infiltrative, extensive nature of the tumor [2,3,7,9]. In cases where complete resection is not feasible or where there is significant local extension, adjuvant radiotherapy is recommended to improve local control. For unresectable tumors, platinum-based chemoradiotherapy is the preferred therapeutic option [7]. Encouraging survival rates and low recurrence have been reported with this approach, challenging the long-standing belief that adenoid cystic carcinoma is radioresistant [11]. Today, the tumor is viewed as radiosensitive, although not truly radio curable, as complete remissions remain rare [9].

In this context, nasopharyngectomy may represent a valuable therapeutic option, particularly for residual or recurrent tumors. However, this procedure remains technically challenging, even with modern skull base surgical techniques, due to the proximity of major neurovascular structures and the limited anatomical access to the nasopharynx. Traditional surgical approaches often provide limited exposure, frequently requiring combined or multi-angled access routes. To overcome these limitations, various endoscopic-assisted techniques have been developed. However, endonasal endoscopic approaches are generally reserved for small tumors, typically stage I or II lesions [12].

In our case, we opted for adaptive radiotherapy using VMAT. The target volumes were carefully delineated to include adjacent cranial nerves up to their emergence at the skull base, given the strong perineural tropism of this type of nasopharyngeal carcinoma.

Additionally, there is growing interest in proton and hadron therapy, particularly for tumors located near critical structures. Thanks to their unique physical and biological properties, these modalities allow for the delivery of higher radiation doses to the tumor while preserving the surrounding healthy tissues [13].

The prognosis for nasopharyngeal adenoid cystic carcinoma is generally considered relatively favorable, largely due to the tumor’s slow progression and low metastatic potential [1]. However, several factors can significantly influence disease outcome. Tumor stage, as well as the presence of cervical lymph node involvement or distant metastases, are among the most important negative prognostic indicators, as they directly impact overall survival [7,8].

Other important risk factors for recurrence include perineural invasion and the nature of the initial treatment, particularly when complete surgical resection or adjuvant radiotherapy is not performed [14]. Therefore, achieving clear surgical margins, combined with adjuvant radiotherapy, significantly reduces the risk of local recurrence. In contrast, advanced-stage tumors, especially those invading the skull base or major vascular structures, tend to have a poorer prognosis, with high rates of recurrence and metastasis typically occurring within the first three years following diagnosis [14]. These cases underscore the importance of close and long-term post-treatment monitoring.

## Conclusions

Adenoid cystic carcinoma of the nasopharynx is a rare tumor, characterized by slow progression, a highly infiltrative nature, a propensity for perineural spread, and a significant risk of local recurrence and distant metastasis. Its management remains challenging and largely depends on tumor stage, locoregional extension, and the feasibility of radical treatment. When surgery is feasible, it remains the preferred approach, typically followed by adjuvant radiotherapy to improve local control. In unresectable cases, modern image-guided radiotherapy may provide meaningful locoregional control while minimizing treatment-related morbidity, as illustrated in this case showing partial local response but subsequent distant pulmonary metastases.

Given the rarity of this disease and the limited data available, multicenter studies would be valuable to better understand prognostic factors and to help standardize treatment strategies.

## References

[1] Liang YF, Kong B, Xiang WY (2014). Nasopharyngeal adenoid cystic carcinoma: a case report and review of the literature. Int J Clin Exp Pathol.

[2] Ng BH, Tang IP (2019). Adenoid cystic carcinoma of the nasopharynx: a case series. Indian J Otolaryngol Head Neck Surg.

[3] Arora V, Yadav V, Mandal G (2021). Adenoid cystic carcinoma of the nasopharynx-a rare entity: our institutional experience and therapeutic approach. Indian J Surg Oncol.

[4] Alsubaie NM, Alhussien A, Alghulikah A, Alabood S, Alsukayt M, Alarifi I (2023). A rare nasopharyngeal adenoid cystic carcinoma: case report and literature review. Am J Case Rep.

[5] Soprani F, Armaroli V, Venturini A, and al (2007). A rare case of adenoid cystic carcinoma of the nasopharynx manifesting as Horner's syndrome: discussion and review of the literature. Acta Otorhinolaryngol Ital.

[6] Aloulou S, Merad-Boudia Z (2002). Adenoid cystic carcinoma (cylindroma) of the nasopharynx with extension into the cavernous sinus (Article in French). Presse Med.

[7] Afani L, Errihani H, Benchafai I, Lalami Y (2016). Nasopharyngeal adenoid cystic carcinoma, a rare but highly challenging disease with unmet therapeutic needs: a case-report and review of the literature (Article in French). Cancer Radiother.

[8] Ko JJ, Siever JE, Hao D, Simpson R, Lau HY (2016). Adenoid cystic carcinoma of head and neck: clinical predictors of outcome from a Canadian centre. Curr Oncol.

[9] Saâdi I, El Marfany M, Hadadi K (2003). Adenoid cystic carcinoma of the nasopharynx: a case report. Cancer Radiother.

[10] Coca-Pelaz A, Rodrigo JP, Bradley PJ (2015). Adenoid cystic carcinoma of the head and neck--an update. Oral Oncol.

[11] Bin-Alamer O, Haider AS, Chaudhary A (2022). Adenoid cystic carcinoma (ACC) infiltrating the skull base: a systematic review of clinical characteristics and management strategies. Cancer Diagn Progn.

[12] Al-Sheibani S, Zanation AM, Carrau RL (2011). Endoscopic endonasal transpterygoid nasopharyngectomy. Laryngoscope.

[13] Hu W, Hu J, Huang Q, Gao J, Zhang H, Kong L (2024). Long-term outcomes after particle radiation therapy in patients with nasopharyngeal adenoid cystic carcinoma. BMC Cancer.

[14] Chen Y, Shi Y, Yu H (2024). Adenoid cystic carcinoma of the nasopharynx: a retrospective study of 12 cases. Ear Nose Throat J.

